# Sub-Frequency Interval Approach in Electromechanical Impedance Technique for Concrete Structure Health Monitoring

**DOI:** 10.3390/s101211644

**Published:** 2010-12-21

**Authors:** Yaowen Yang, Bahador Sabet Divsholi

**Affiliations:** School of Civil and Environmental Engineering, Nanyang Technological University, 50 Nanyang Avenue, 639798, Singapore; E-Mail: sabe0002@ntu.edu.sg

**Keywords:** piezoelectric, Electromechanical Impedance (EMI), concrete, Structural Health Monitoring (SHM)

## Abstract

The electromechanical (EM) impedance technique using piezoelectric lead zirconate titanate (PZT) transducers for structural health monitoring (SHM) has attracted considerable attention in various engineering fields. In the conventional EM impedance technique, the EM admittance of a PZT transducer is used as a damage indicator. Statistical analysis methods such as root mean square deviation (RMSD) have been employed to associate the damage level with the changes in the EM admittance signatures, but it is difficult to determine the location of damage using such methods. This paper proposes a new approach by dividing the large frequency (30–400 kHz) range into sub-frequency intervals and calculating their respective RMSD values. The RMSD of the sub-frequency intervals (RMSD-S) will be used to study the severity and location of damage. An experiment is carried out on a real size concrete structure subjected to artificial damage. It is observed that damage close to the PZT changes the high frequency range RMSD-S significantly, while the damage far away from the PZT changes the RMSD*-*S in the low frequency range significantly. The relationship between the frequency range and the PZT sensing region is also presented. Finally, a damage identification scheme is proposed to estimate the location and severity of damage in concrete structures.

## 1. Introduction

With the reported increasing number of collapses occurring in major infrastructures, health monitoring of civil structures has become of significant importance. Structures must satisfy strength and serviceability criteria throughout their stipulated design life. However, after a natural disaster, such as an earthquake, the strength and serviceability of the structure may become questionable due to possible damage induced in it. In addition, to prevent catastrophic failures, gradual deterioration of structures with time, environmental corrosion, lack of maintenance, accidental overloading and excessive usage all require periodic evaluation during the life span of the structures.

Concrete structures have been used extensively in civil infrastructural systems. However, compared to metallic or other composite structures, non-destructive evaluation (NDE) technologies of concrete structures are relatively undeveloped [1]. Many types of structural health monitoring (SHM) techniques have been reported in the literature, based on either the global or the local monitoring of structures [2]. Some researchers have proposed methods based on the global static response of structures, such as the static displacement response technique [3] and the static strain measurement technique [4]. The main limitation of the static response techniques is that their application on large structures is not feasible in practice. The static displacement technique involves applying static forces to the structure at specific nodal points and measuring the corresponding displacements which is expensive and tedious task for real size structures. In global dynamic techniques, the structure is subjected to low frequency excitations and the first few mode shapes and their corresponding natural frequencies are extracted. The main drawback of these techniques is that they rely on a small number of low order modes, which, being global in character, are not very sensitive to the presence of localized damages. Another limitation of these techniques is interference from ambient vibration noise, which happens to be in the low frequency range, typically less than 100 Hz [2].

Another category of damage detection techniques are the so-called local methods, which rely on the localized monitoring of the structures. Some common techniques in this category are the ultrasonic wave propagation technique, acoustic emission, magnetic field analysis, electrical methods, dye penetrant testing, impact echo testing and X-ray radiography [5]. However, these techniques share many drawbacks which prevent their use for health monitoring of large civil structures [2,6,7].

Due to the increasing number of infrastructures and the need of monitoring inaccessible areas, manual monitoring becomes less interesting and not applicable for most projects. In fact, some of the important structures such as dams and nuclear stations need online continuous monitoring due to potentially disastrous nature of any failure. Online damage detection systems would reduce costs by minimizing maintenance and inspection cycles. One of the most promising means of developing such systems is through the integration of smart materials such as piezoelectric materials into the structures under monitoring.

The piezoelectric ceramic lead zirconate titanate (PZT)-based electromechanical impedance (EMI) technique is a very promising technique for SHM. In the EMI method, the electromechanical (EM) admittance signatures of the PZT acquired at different time are used to calculate the damage indicator [8]. The one dimensional EMI model was developed as follows [9]:

(1) $$\bar{Y}=(\omega j)\frac{wl}{h}[({\bar{ɛ}}_{33}^{T}-d_{31}^{2}\bar{E})+(\frac{Z_{a}}{Z+Z_{a}})d_{31}^{2}\bar{E})(\frac{\text{tan}\;\kappa l}{\kappa l})]$$

where *w*, *l* and *h* are the width, length and height of the PZT, respectively; *Z_a_* is the short circuit mechanical impedance of PZT and *Z* is the mechanical impedance of a structure; *Ē* = *E*(1+*ηj*) is the complex modulus of elasticity and ${\bar{ɛ}}_{33}^{T}={ɛ}_{33}^{T}(1-\delta j)$ is the complex electric permittivity at constant stress and $j=\sqrt{-1}$; η and δ are the mechanical loss factor and dielectric loss factor, respectively; *d*_31_ is the piezoelectric constant; κ is the wave number, related to angular frequency of excitation *ω* by $\kappa =\omega \sqrt{\rho /\bar{E}}$, where ρ is density of PZT. The electromechanical admittance signatures *Ȳ* consists of a real part (the conductance) and an imaginary part (the susceptance). Conductance has been traditionally used for structural health monitoring due to its better indication of structural changes [10]. The prominent effects of structural damage or material characteristic change on the PZT admittance signatures are the lateral and vertical shifting of the baseline or appearance of new peaks in the signatures, which are the main indicators of damages or material changes [11].

Statistical techniques such as root mean square deviation (RMSD) [6] have been employed to associate the damage or material changes with the changes in the PZT admittance signatures:

(2) $$\text{RMSD}(\%)=\sqrt{\frac{\sum_{i=1}^{N}{\left(G_{i}^{1}-G_{i}^{0}\right)}^{2}}{\sum_{i=1}^{N}{\left(G_{i}^{0}\right)}^{2}}}\times 100$$

where $G_{i}^{0}$ is the baseline signature of PZT conductance; and $G_{i}^{1}$ is the corresponding conductance for each monitoring time at the *i^th^* measurement point. Generally, larger difference between the baseline signature and the subsequent signatures would result in bigger RMSD values.

The principle behind this technique is to apply high-frequency structural excitations (typically higher than 30 kHz) through surface-bonded PZT transducers, and measure the impedance of structures. Park *et al*. [8] recommended a frequency range from 30 kHz to 400 kHz for PZT patches. To achieve high sensitivity to damage, high frequency signatures (>200 kHz) have been used to monitor the region close to the PZT location, while low frequency signatures (<100 kHz) have been traditionally ignored. However, it has been reported that in the use of the electromechanical impedance technique with RMSD as the damage indicator it is difficult to specify the damage location and severity. Wave propagation techniques in the time domain were proposed to locate damage in structures [8].

In the EMI technique, the elastic waves generated by the PZT transducer propagate in the structure. The waves are reflected back to the PZT transducer when they encounter some geometric discontinuity or damage in the structure. The reflected wave thus contains the vital information about any structural damage, which can be recorded by the impedance analyzer in the PZT admittance signature in the frequency domain. The attenuation of waves is very high in concrete structures in comparison to metallic structures due to their higher porosity and inconsistent nature. In addition, the attenuation of waves increases with the frequency of monitoring. Thus, higher frequency results in smaller sensing regions. However, no attempt has been made to quantitatively correlate the frequency range with the sensing region in the concrete. This paper proposes a new approach by dividing the large frequency (30–400 kHz) range into sub-frequency intervals and calculating their respective RMSD values. Instead of the single value of RMSD used in the previous EMI methods, the RMSD of sub-frequencies (RMSD-S) will be used to study the severity and location of damage. An experiment is carried out on a real size concrete structure subjected to artificial damages. The PZT admittance signatures in a frequency range of 30 to 400 kHz for various structural damages have been recorded and the RMSD-S values are calculated. It is observed that the damage close to the PZT changes the RMSD-S of the high frequency range significantly, whereas the damage far away from the PZT changes the RMSD-S of the low frequency range significantly. The relationship between the frequency range and the PZT sensing region is also presented. Finally, a damage identification scheme is proposed to estimate the location and severity of damage in concrete structures.

## 2. Experimental Work

**Figure 1.**
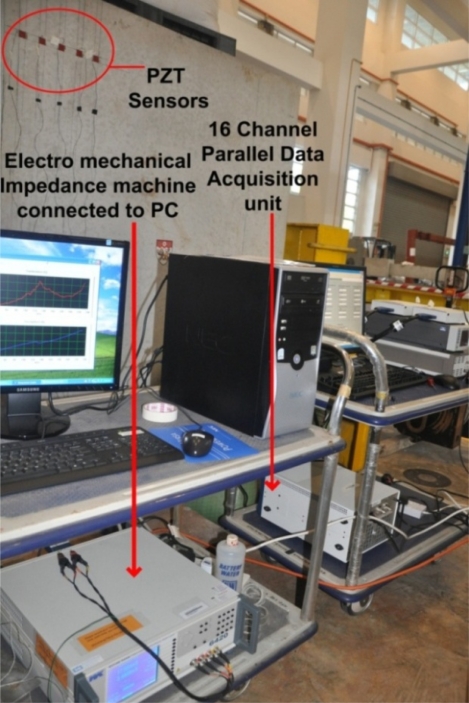
Experimental setup.

Five 20 × 20 × 0.5 mm PZTs were attached to a concrete mass of 2.0 × 1.5 × 1.5 m. A two-component adhesive (3M’s DP 460 epoxy) was used to bond the PZTs to the surface of the concrete. The specifications and physical properties of the PZT patches can be obtained from PI Ceramic [12]. A waterproofing agent called Plastic DIP [13] was used to cover the exposed surface of PZTs to prevent deterioration of the transducers over time. An impedance analyzer with maximum voltage level of 2 volt and a multiplexer were used to acquire the electromechanical admittance signatures of the PZTs. A 16 channel parallel data acquisition unit was used to study the PZT generated wave propagation on the surface of concrete structure. Figure 1 shows the overall experimental setup.

**Figure 2.**
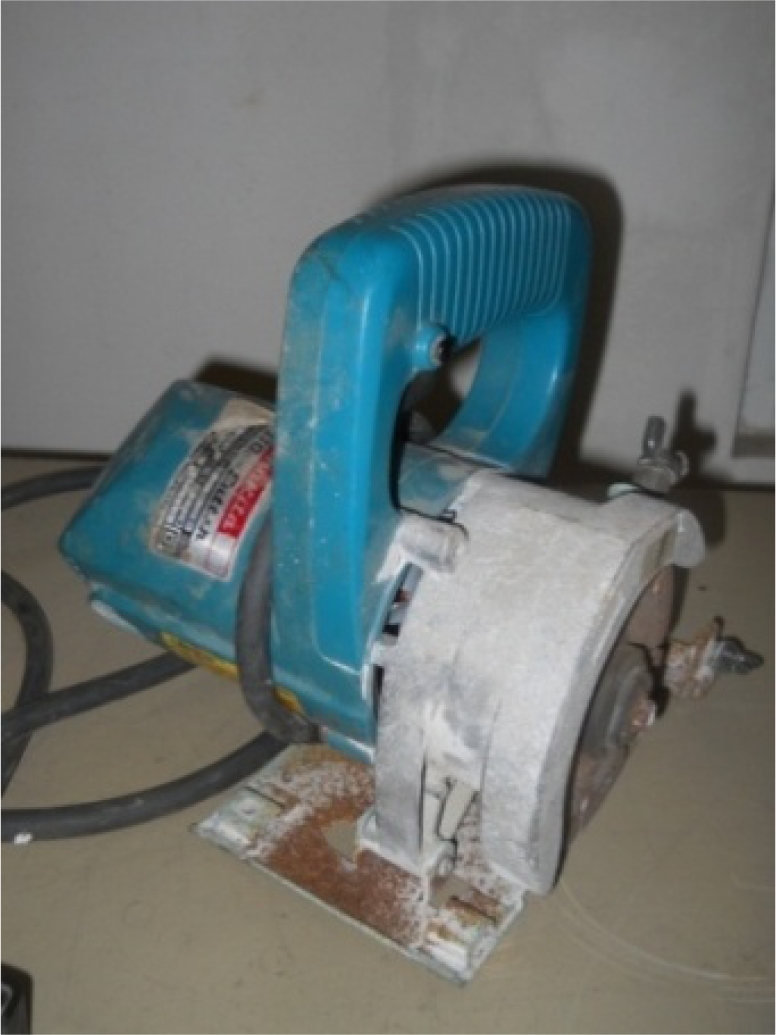
Circular saw used to create the damages.

**Figure 3.**
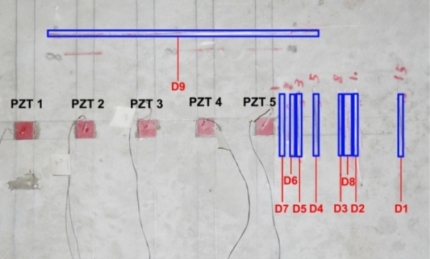
Location and sequence of damages on the structure.

In our earlier work [14], a drill hammer was used to create hole-type damages in the concrete structure. However, it was found that lots of hairline cracks formed near the holes, which caused some uncertainties in the results. To avoid this problem, in this study, the hand-held circular saw shown in Figure 2 was used to create crack-type damages in the concrete. Figure 3 shows the location and sequence of damages.

**Figure 4.**
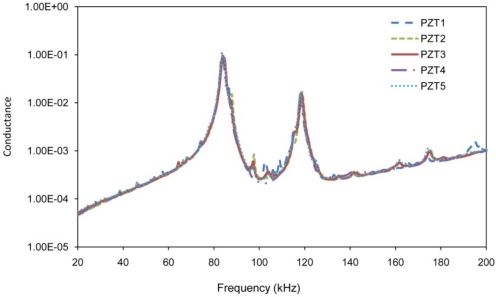
Free signatures of the PZT transducers.

Free signatures of PZT transducers acquired before attaching them to the structure are presented in Figure 4, which shows the good repeatability.

## 3. Results and Discussion

### 3.1. Sensing Region

PZT transducers have been used for monitoring of structures based on two principles: (1) using multiple PZTs in the wave propagation technique, and (2) using a single PZT transducer in the EMI technique or the pulse-echo technique. The wave propagation technique is similar to the conventional ultrasonic technique. One PZT transducer is used to generate high frequency waves with constant frequency in a structure; one or more PZT transducers acquire the signal. Then, the acquired signal in time domain is investigated to assess the damage. Usually, finding the location of damage demands complex modeling of wave propagation.

**Figure 5.**
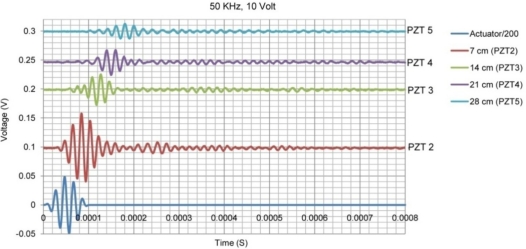
Wave propagation at frequency of 50 kHz with PZT 1 as actuator.

**Figure 6.**
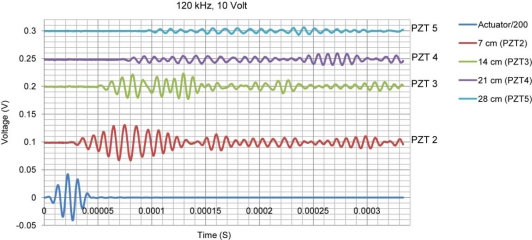
Wave propagation at frequency of 120 kHz with PZT 1 as actuator.

The wave velocity changes in concrete with frequency [15]. In addition, the wave modes and velocity change as it hits boundaries. Figure 5 shows the wave propagation signals at 50 kHz frequency with an actuation voltage of 10 volt applied on PZT 1. PZT 1 was used as actuator and other PZTs as sensors. Figure 6 shows the same signals but at 120 kHz frequency.

**Figure 7.**
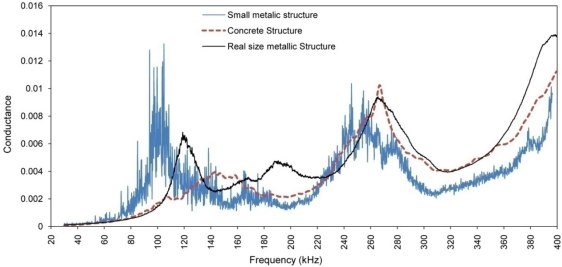
Typical EMI signature for small metallic structures, concrete structure and real size metallic structure.

Comparison between the acquired signals in Figures 5 and 6 reveals obvious changes in speed and amplitude of the signals. Higher attenuation of wave at frequency of 120 kHz is also observed in comparison to that at 50 kHz for distances far away from the PZT (in this case comparing the signal received by PZTs 4 and 5 in Figures 5 and 6). In the EMI technique, similar to the plus-echo technique, one PZT transducer is used as both actuator and sensor. However, instead of studying the wave propagation signal at one particular frequency in the time domain, the EMI focuses on the admittance signal in the frequency domain obtained by sweeping the signal in a pre-selected frequency range. At each frequency, the PZT transducer which is controlled by the impedance analyzer generates a steady state wave propagating through the structure and receives the reflected wave signal in the form of EM admittance. For each frequency one value of admittance is measured. If there is no change in structure, this value will remain the same in repetitive measurements. The impedance analyzer generates a high frequency signal with voltage level of one or two volt. In health monitoring of small metallic structures, the generated wave is strong enough to cover the whole structure, activate the vibration modes and locate their corresponding natural frequencies. Figure 7 shows a typical EMI signature for an aluminum beam structure of 30 × 5 × 0.2 cm size.

PZT generated waves will be attenuated when propagating from the PZT to a structure through an epoxy layer, propagating in the structure and returning to the PZT. For real size metallic structures or medium size concrete structures, the generated waves cannot activate the modal vibration of the structure due to insufficient energy in actuating signal. Thus, a PZT only monitors a limited area around itself. While the EM signature contains the local dynamic information of the structure, the major peaks in the signature do not represent the natural frequencies of the structure. Instead, they represent the PZT resonant frequencies, as shown in Figure 7. In addition, wherever waves hit any boundary or inconsistency such as cracks, they will be reflected back to the PZT.

**Figure 8.**
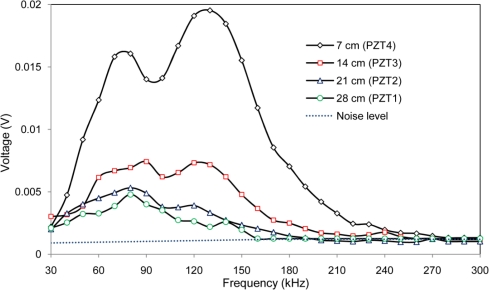
Maximum voltage received from PZT 1 to 4 by setting PZT 5 as actuator.

Figure 8 shows the maximum voltage received by PZTs 1 to 4 by setting PZT 5 as actuator with a voltage level equal to 2 volt, which represents the current maximum voltage in the impedance analyzer used in this work. Figure 8 reveals very useful information about the sensing region of PZT transducers for health monitoring of concrete structures. It is noted that a negligible signal was received by the PZT 28 cm away from the actuator for frequencies above 160 kHz. Similarly, a weak signal was received from the PZTs at 21 cm, 14 cm and 7 cm for frequencies above 190 kHz, 230 kHz and 270 kHz, respectively. This means that when the monitoring frequency increases, the sensing region decreases. In the EMI technique, the sensing distance is half of these values as the wave needs to travel and return to the same PZT as it serves as both actuator and sensor. The major peaks near 80 kHz and 120 kHz correspond to the natural frequencies of PZT traducers shown in Figure 4.

Total induced force of PZT transducer can be calculated as follows [16]:

(3) $$F=\bar{E}whl({ɛ}_{p}-d_{31}\frac{v}{h})$$

where *ɛ_p_* is the free strain inside PZT and V is the applied voltage. It can be seen that the generated force is proportional to the applied voltage, the length and the width of PZT. Increasing the voltage from 2 to 10 volt amplifies the received signal exactly five times. The sensing region depends on monitoring frequency range, size of PZT, concrete material properties and monitoring voltage [17]. The attenuation of wave for various frequency ranges can be studied by considering the results presented in Figure 8 or other literature results [15,18]. Therefore, the sensing region can be determined and controlled by considering these parameters.

### 3.2. Damage Detection

**Figure 9.**
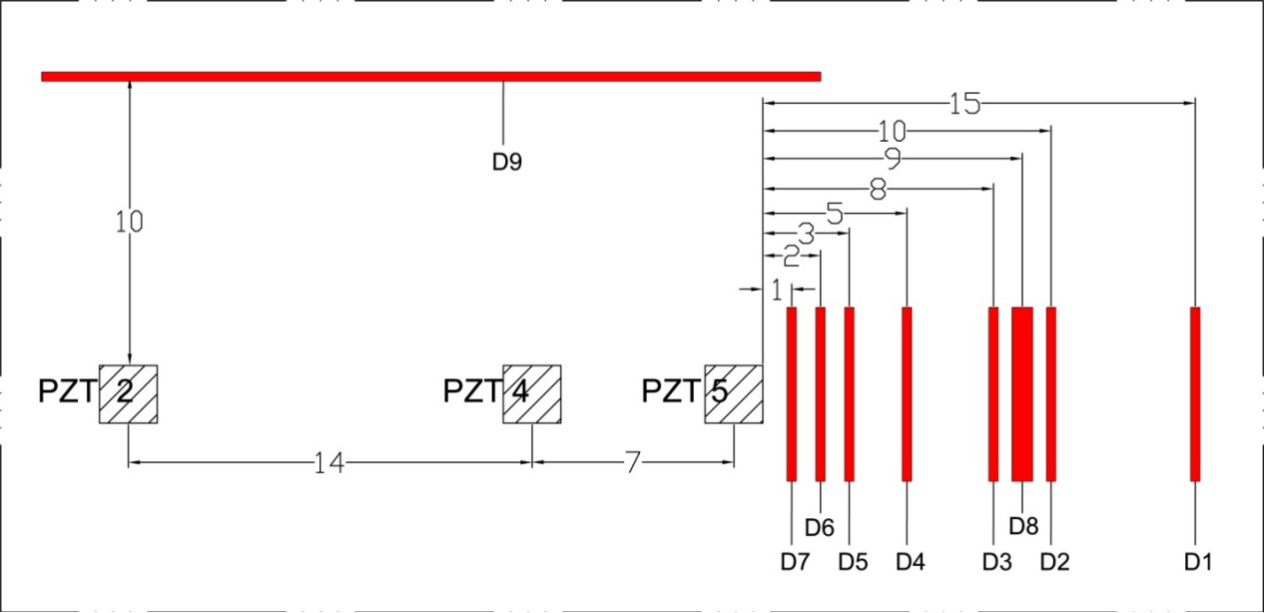
Location and sequence of damages for EMI method.

As mentioned earlier, an impedance analyzer with maximum voltage of 2 volt was used to acquire the EM admittance signature of the structure. The monitoring range was selected from 30 to 400 kHz with a stepping frequency of 0.1 kHz. Figure 9 shows the location and sequence of damages and PZT locations, where D1 represents the first damage and D9 the last damage. Tables 1, 2 and 3 present the RMSD-S results of the admittance signatures acquired after each damage for PZTs 5, 4 and 2, respectively.

**Figure 10.**
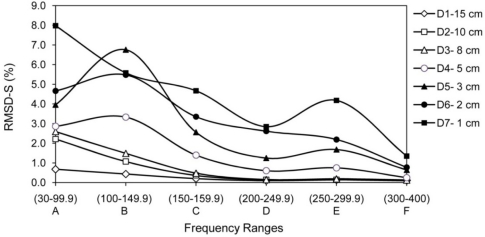
RMSD-S of damage D1 to D7 for PZT 5.

The signature obtained under the healthy condition was considered as the baseline for damage D1; the signature acquired after damage D1 was considered as the baseline for damage D2. That is, the signature after one damage was used as the baseline for the next one. The whole monitoring frequency range of 30–400 kHz is divided into six sub-frequency intervals, *i.e.*, (A) 30–99.9 kHz, (B) 100–149.9 kHz, (C) 150–199.9 kHz, (D) 200–249.9 kHz, (E) 250–299.9 kHz and (F) 300–400 kHz. The RMSD values for the entire frequency range and the sub-frequency intervals (RMSD-S) are calculated for each damage state. Figure 10 shows the RMSD-S of damage D1 to D7 in 15 cm, 10 cm, 8 cm, 5 cm, 3 cm, 2 cm and 1 cm from PZT 5, respectively. For the damages far away from PZT 5, namely D1, D2 and D3, the low frequency range (A) shows more changes compared to the other frequency ranges. These results are expected as the high frequency waves cannot travel and return to PZT 5 from D1, D2 and D3 due to the longer distance for wave travel. In other words, the high frequency signals are not able to sense those damages far away from the PZT. As the damages D4, D5 and D6 are getting closer to PZT 5, the mid range RMSD-S for intervals (B), (C) and (D) show more sensitive changes. There is a clear increase in RMSD-S compared to earlier cases for damage D7 at 1 cm for frequency range (E) which can monitor a very small region close to PZT.

**Figure 11.**
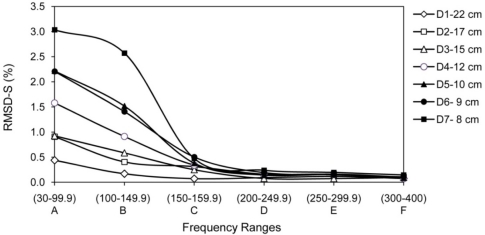
RMSD-S of damage D1 to D7 for PZT 4.

Figure 11 shows the RMSD-S of damages D1 to D7 for PZT 4. D1 to D7 are at 22 cm, 17 cm, 15 cm, 12 cm, 10 cm, 9 cm and 8 cm from PZT 4, respectively. Again, the results agree with the expectation. D1 to D4 are far way from PZT 4 and only range (A) exhibits more sensitive changes. For damages D5 to D7 which are closer to PZT 4, ranges (B) and (C) show more sensitivity as well. However, as expected, the high frequency ranges (D) to (F) cannot pick up the damages.

**Figure 12.**
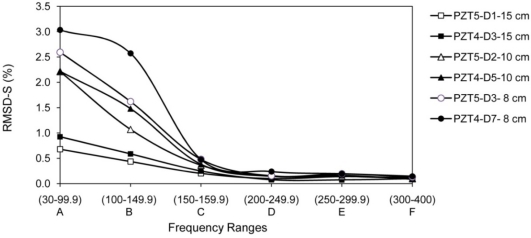
RMSD-S for PZTs 4 and 5 for similar distance to damage.

Damages D3, D5, D7 are at 15 cm, 10 cm and 8 cm from PZT 4, respectively. Damage D1, D2 and D3 are also at 15 cm, 10 cm and 8 cm from PZT 5. Thus, the RMSD-S for these cases are compared in Figure 12. Damages are not identical and result in different amount of change in RMSD-S values, however patterns are very repeatable, which shows the potential of the proposed method. Damage D8 is created between D2 and D3, the objective is to investigate the applicability of proposed method in presence of existing damages. As shown in Table 1 column D8, only low frequency range shows significant change in RMSD-S. However the values are smaller compare to RMSD-S of damages D2 and D3 due to the interference from the existing damages which increases the wave attenuation. Damage D9 was created on top of the three PZTs in distance of 10 cm. As the distance is same, three PZTs showed very good repeatability in the RMSD-S results as shown in Table 1 to 3, column D9.

**Figure 13.**
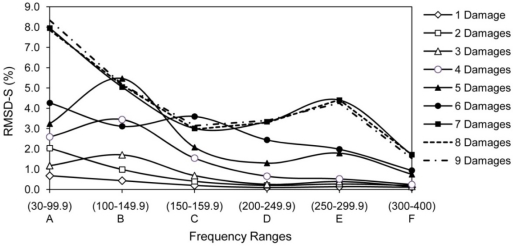
RMSD-S for cumulative effect of damages for PZT 5.

Analyzing the effect of cumulative damages is useful and may produce consistent results in RMSD-S. In this case, the baseline is set as healthy state and all the signatures are compared with the healthy signature. Figure 13 shows the RMSD-S for cumulative effect of one to nine damages. Based on the location of damages, four separate patterns of RMSD-S for (1) one and two damages, (2) three, four and five damages, (3) six damages and (4) seven damages can be identified. As expected, addition of D8 and D9 did not change the pattern as they both occurred in far away from location of PZT 5. Comparing with one common baseline can be used as alternative way in analysis of RMSD-S results.

### 3.3. Comparison of RMSD-S with RMSD

**Figure 14.**
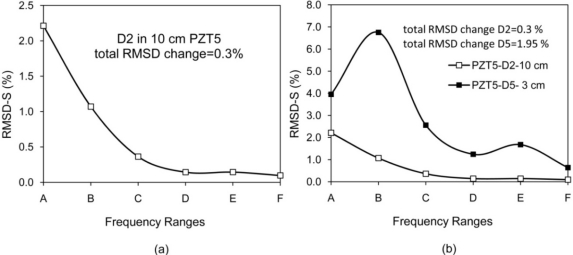
Possible issues of using RMSD as single value instead of RMSD-S, (a) Damage occurred far away from PZT (b) Similar damage occurred at two different distances from PZT.

Considering RMSD-S instead of RMSD can reduce uncertainties in damage identification. For instance, in the case of damage D2 (at 10 cm from PZT 5) in Table 1, RMSD-S for range (A) changes 2.2%, but the total RMSD changes 0.3% and the RMSD-S for higher frequencies (>200 kHz) changes less than 0.15%. This damage could be overlooked if a limited high frequency range is monitored. Figure 14(a) illustrates this problem. In other words, if a major crack exists at that distance, it may change the RMSD-S for range (A) significantly, but the total RMSD and RMSD-S for higher frequency ranges may only change slightly. The occurrence of major crack on that distance could be measured by considering RMSD-S. Similar damages in two different distances from PZT may change the RMSD-S with different patterns. The damages D2 and D5 are comparable in size but located at 10 cm and 3 cm from PZT5. Total RMSD increased by 0.3 % and 1.95 %, respectively. These numbers do not indicate whether the bigger damage at the same or a similar distance or a smaller damage closer to the PZT has occurred. Yan *et al*. [19] employed the covariance (COV) index to locate damages in a Timoshenko beam with distributed PZT patches. However, similar to the RMSD index, the COV index has difficulty in distinguishing the EMI signatures between a bigger damage closer to the PZT and a smaller damage far away from the PZT. Considering RMSD-S is a suitable approach to solve this problem. For damages closer to the PZT, in this case D5 at 3 cm, the higher frequency shows more sensitive changes. Figure 14(b) illustrates this problem.

In addition, selecting the limited low frequency range may not be suitable for damage identification. Both severe damage far away and small damage close to PZT may change the RMSD for low frequency significantly. A possible way to distinguish between them is looking at the RMSD-S at high frequency ranges simultaneously. Damage D7 which is at 1 cm from PZT5 changes the RMSD-S for range (A) significantly, a major crack far away from PZT5 could change the RMSD of range (A) by similar amount. For this case, considering RMSD-S can reveal whether a small damage close to PZT or major damage far away from PZT is occurred. The damage close to PZT (1 cm) would change the RMSD-S at high frequency significantly compare to damage far away from PZT which cannot be sensed by high frequency ranges.

**Figure 15.**
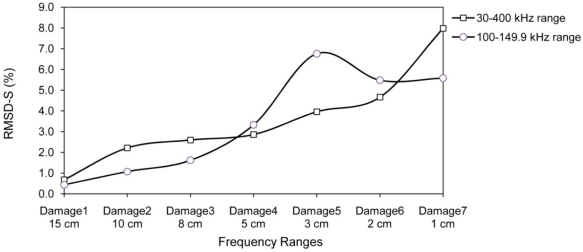
Inconsistency in EMI result for RMSD of limited frequency range.

Figure 15 reveals another limitation of considering limited frequency range. For frequency range of 100 kHz to 150 kHz, the RMSD value for damage D5 at 3 cm from PZT5 is higher than damages D6 and D7 at 2 cm and 1 cm from PZT5, respectively. In addition, no information on location and severity of these damages can be obtained.

**Figure 16.**
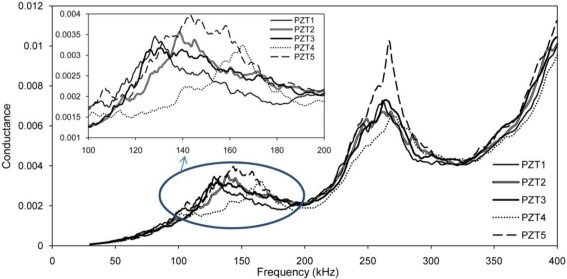
PZT signatures in healthy condition.

**Figure 17.**
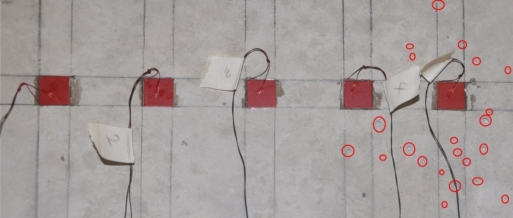
Surface condition near PZTs.

As mentioned earlier, for real size metallic and concrete structures, each PZT monitors limited area around itself and based on surface and boundary condition near that PZT, the signature would be different. Figure 16 shows the EMI signature obtained from five PZTs before inducing any damage. Signatures acquired from PZT 4 and 5 are varied significantly from three others. Figure 17 shows the surface condition around these PZTs. The concrete surface was more porous close to PZT 4 and 5.

## 4. Damage Identification Scheme

**Figure 18.**
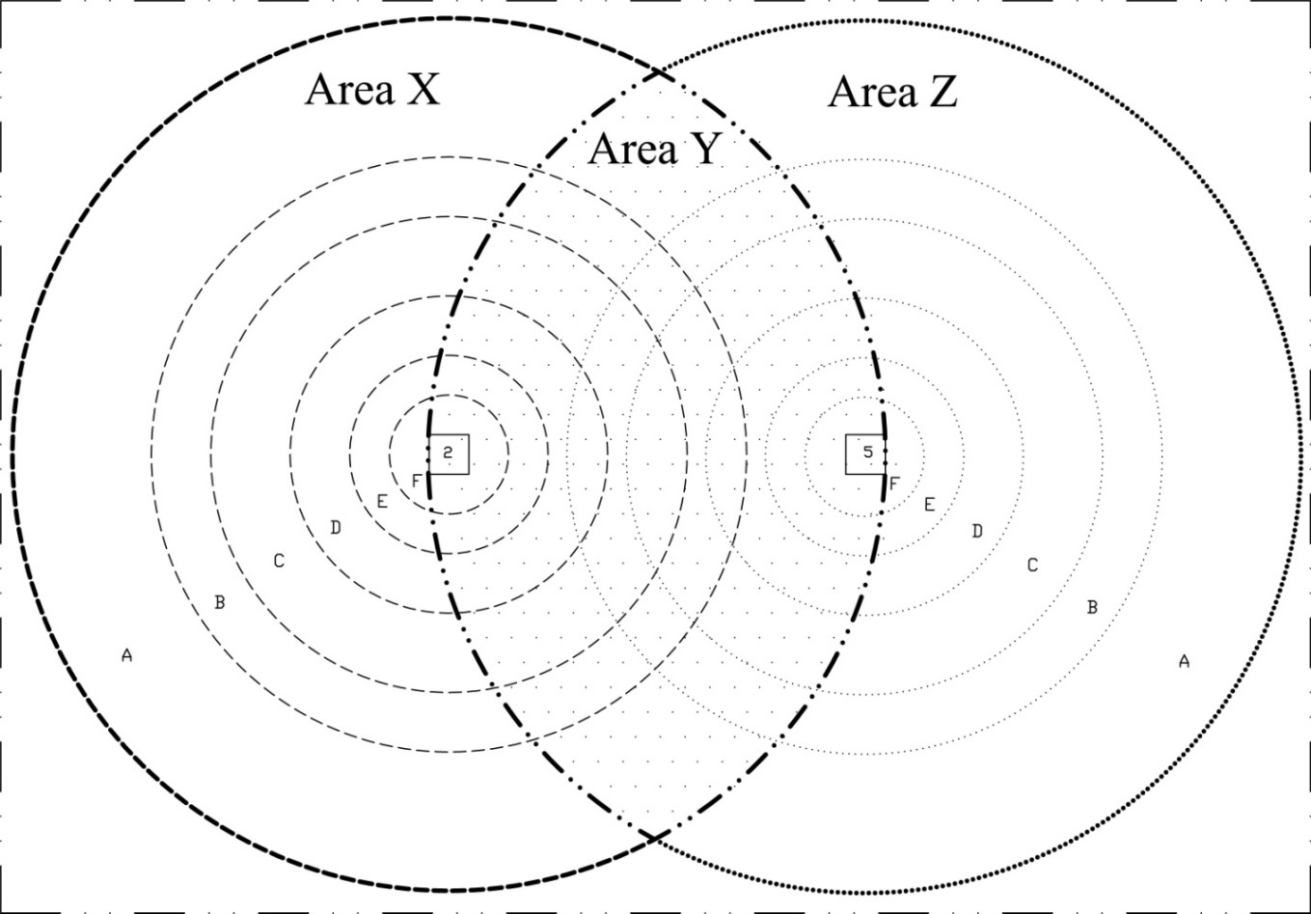
Selected area of concrete wall.

Given the RMSD-S results, a method is presented here to investigate the location and severity of damage. Consider a section of the above concrete structure in Figure 18, where two PZTs 2 and 5 with distance of 21 cm are used for monitoring. Table 4 shows the RMSD-S results versus distance of damage from PZT for three PZTs. For each frequency range, the RMSD-S values which changed noticeably are highlighted in the table. Considering the results presented in Table 4, the radius of sensing regions for the six frequency ranges of (A) to (F) can be determined as 22 cm, 15 cm, 12 cm, 8 cm, 5 cm and 3 cm, respectively. A slightly larger sensing region of 30 cm was reported in [20] due to the use of lower frequency range of 20–25 kHz.

**Figure 19.**
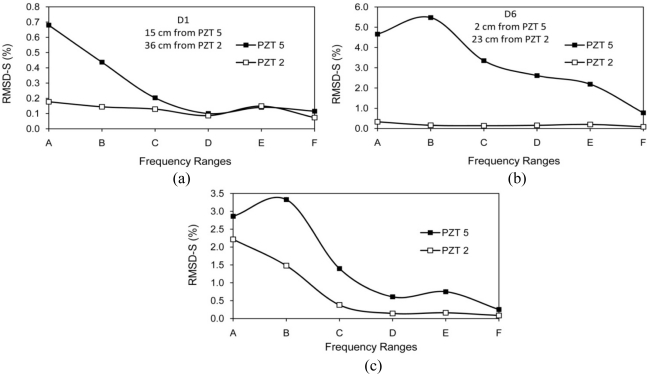
Predicted location of damage for various RMSD-S, (a) Damage in area (A) of region Z, (b) Damage in area (E) or (F) of region Z, (c) Damage in region Y in area (A) or (B) for PZT 2 which overlaps with area (C) to (E) for PZT 5.

The six sensing regions (A) to (F) are illustrated using circles for both PZTs 2 and 5 in Figure 18. The monitoring area can be divided into three main regions, the left hand side of PZT 2 (Region X), right hand side of PZT 5 (Region Z) and the region between PZTs 2 and 5 (Region Y). If any damage occurs in Region X, only PZT 2 can sense it. Similarly, damages in Region Z can only be detected by PZT 5, and damages in Region Y can be detected by both PZTs. It is therefore possible to estimate the location of damage from the RMSD-S values of two PZTs. Three cases with different RMSD-S values for two PZTs are presented in Figure 19. The RMSD-Ss are based on actual damages in Tables 1 and 3. In Case 1 presented in Figure 19(a), the RMSD-S of PZT 2 is too small (around 0.2%) to sense the damage. Thus the damage should be outside the sensing region of PZT 2 and located in Region Z. The RMSD-S of PZT 5 is noticeable (around 0.7%) in low frequency range (A), indicating that the damage is in area (A) in Region Z. In Case 2 presented in Figure 19(b), the RMSD-S of PZT 2 is very small and that of PZT 5 varies from 5% to 0.8% in frequency range (A) to (F). Thus, the damage is estimated in Region Z and close to PZT 5 in area (E) or (F). In Case 3 presented in Figure 19(c), the RMSD-S of both PZTs is noticeable in low frequency ranges. Thus the damage is in region Y between the two PZTs. The RMSD-S of PZT 5 is changed more significantly for higher frequencies. Therefore the damage is closer to PZT 5 in area (C) to (E) which overlaps with area (A) or (B) of PZT 2. One of the applications of above scheme is to monitor remote areas such as column-beam joints. In column-beam load transfer system, safety of joint is very important. In the above illustration, by using two pieces of PZTs with maximum sensing region of 22 cm, an area of 66 × 44 cm can be monitored. Using more PZTs or decreasing the distance between PZTs will help to estimate the location of damages with higher accuracy.

## 5. Conclusions

In this work, a sub-frequency interval approach RMSD-S has been proposed to analyze the electromechanical impedance (EMI) signature of piezoelectric transducers. Compared with the conventional approach which uses one RMSD value for the entire frequency range, the proposed technique is more reliable and robust for health monitoring of concrete structures. The damage severity and location can be estimated by a single PZT sensor through the RMSD-S. As shown in Section 4, multiple PZTs can increase the accuracy of damage identification and provide a larger sensing area. It is observed that the damage close to PZT changes the RMSD-S at high frequency range significantly; however the damage far away from the PZT changes the low frequency range RMSD-S significantly. A damage identification scheme has been proposed to study the location and severity of damage in concrete structures.

## Figures and Tables

**Table 1.** RMSD-S for PZT 5.

	D 1	D 2	D 3	D 4	D 5	D 6	D 7	D 8	D 9
Distance (cm)	15	10	8	5	3	2	1	9	10
Frequency (kHz)									
30–400	0.16	0.30	0.41	0.95	1.95	2.11	3.12	0.18	0.47
(A) 30–99.9	0.68	2.21	2.59	2.86	3.96	4.66	7.98	1.14	2.58
(B) 100–149.9	0.44	1.07	1.75	3.33	6.76	5.48	5.58	0.38	1.55
(C) 150–199.9	0.20	0.36	0.48	1.40	2.56	3.35	4.67	0.24	0.67
(D) 200–249.9	0.10	0.14	0.22	0.61	1.24	2.61	2.84	0.18	0.21
(E) 250–299.9	0.14	0.14	0.19	0.75	1.68	2.19	4.18	0.19	0.38
(F) 300–400	0.12	0.10	0.13	0.23	0.64	0.77	1.35	0.10	0.11

**Table 2.** RMSD-S for PZT 4.

	D 1	D 2	D 3	D 4	D 5	D 6	D 7	D 8	D 9
Distance (cm)	22	17	15	12	10	9	8	16	10
Frequency (kHz)									
30–400	0.11	0.17	0.16	0.23	0.33	0.33	0.51	0.12	0.36
(A) 30–99.9	0.44	0.91	0.93	1.58	2.22	2.21	3.04	0.38	2.39
(B) 100–149.9	0.17	0.40	0.59	0.91	1.52	1.41	2.40	0.18	1.48
(C) 150–199.9	0.07	0.31	0.22	0.34	0.38	0.50	0.47	0.19	0.69
(D) 200–249.9	0.09	0.16	0.08	0.15	0.14	0.22	0.27	0.12	0.22
(E) 250–299.9	0.12	0.16	0.07	0.15	0.16	0.15	0.22	0.13	0.15
(F) 300–400	0.08	0.10	0.10	0.07	0.08	0.11	0.14	0.08	0.09

**Table 3.** RMSD-S for PZT 2.

	D 1	D 2	D 3	D4	D 5	D6	D7	D 8	D 9
Distance (cm)	36	31	29	26	24	23	22	30	10
Frequency (kHz)									
30–400	0.10	0.11	0.09	0.06	0.07	0.14	0.12	0.12	0.26
(A) 30–99.9	0.18	0.19	0.24	0.27	0.37	0.33	0.34	0.19	2.13
(B) 100–149.9	0.14	0.08	0.06	0.08	0.11	0.16	0.12	0.11	0.75
(C) 150–199.9	0.13	0.12	0.04	0.05	0.06	0.14	0.12	0.13	0.43
(D) 200–249.9	0.09	0.08	0.09	0.06	0.07	0.16	0.12	0.12	0.16
(E) 250–299.9	0.15	0.08	0.06	0.04	0.07	0.20	0.17	0.14	0.13
(F) 300–400	0.07	0.12	0.11	0.05	0.05	0.08	0.09	0.12	0.10

**Table 4.** RMSD-S versus distance for three PZTs.

Distance of damage from PZT (cm)	Frequency Range (kHz)						
	30–400	(A) 30–99.9	(B) 100–149.9	(C) 150–199.9	(D) 200–249.9	(E) 250–299.9	(F) 300–400
1	3.12	**7.98**	**5.58**	**4.67**	**2.84**	**4.18**	**1.35**
2	2.11	**4.66**	**5.48**	**3.35**	**2.61**	**2.19**	**0.77**
3	1.95	**3.96**	**6.76**	**2.56**	**1.24**	**1.68**	**0.64**
5	0.95	**2.86**	**3.33**	**1.40**	**0.61**	**0.75**	0.23
8	0.51	**3.04**	**2.57**	**0.47**	**0.27**	0.22	0.14
10	0.33	**2.22**	**1.52**	**0.38**	0.14	0.16	0.08
12	0.23	**1.58**	**0.91**	**0.34**	0.15	0.15	0.07
15	0.16	**0.93**	**0.59**	0.22	0.08	0.07	0.10
22	0.11	**0.44**	0.17	0.07	0.09	0.12	0.08
29	0.09	0.24	0.06	0.04	0.09	0.06	0.11
31	0.11	0.19	0.08	0.12	0.08	0.08	0.12
36	0.10	0.18	0.14	0.13	0.09	0.15	0.07
